# A New Case of 13q12.2q13.1 Microdeletion Syndrome Contributes to Phenotype Delineation

**DOI:** 10.1155/2014/470830

**Published:** 2014-11-23

**Authors:** Giorgia Mandrile, Eleonora Di Gregorio, Alessandro Calcia, Alessandro Brussino, Enrico Grosso, Elisa Savin, Daniela Francesca Giachino, Alfredo Brusco

**Affiliations:** ^1^Medical Genetics Unit, “San Luigi Gonzaga” University Hospital, University of Torino, Regione Gonzole 10, 10143 Orbassano, Italy; ^2^Department of Clinical & Biological Sciences, University of Torino, 10143 Orbassano, Italy; ^3^Department of Medical Sciences, University of Torino, Via Santena 19, 10126 Torino, Italy; ^4^Medical Genetics, “Città della Salute e della Scienza” University Hospital, 10126 Torino, Italy

## Abstract

A recently described genetic disorder has been associated with 13q12.3 microdeletion spanning three genes, namely, *KATNAL1, LINC00426*, and *HMGB1*. Here, we report a new case with similar clinical features that we have followed from birth to 5 years old. The child carried a complex rearrangement with a double translocation: 46,XX,t(7;13)(p15;q14),t(11;15)(q23;q22). Array-CGH identified a *de novo* microdeletion at 13q12.2q13.1 spanning 3–3.4 Mb and overlapping 13q12.3 critical region. Clinical features resembling those reported in the literature confirm the existence of a distinct 13q12.3 microdeletion syndrome and provide further evidence that is useful to characterize its phenotypic expression during the 5 years of development.

## 1. Introduction

Array-CGH has gained increased recognition as a first-tier technique to identify and characterize the genetic determinants of intellectual disability (ID) syndromes. Following its introduction, the detection rate of molecular cytogenetic alterations has increased by up to 15% in unselected patients with ID [1, 2]. The possibility of comparing patient phenotypes with overlapping rearrangements has led to the identification of several novel microdeletion/microduplication syndromes [2]. A 13q12.3 microdeletion syndrome has recently been described involving a ~300 kb critical region spanning only three genes, namely,* KATNAL1*,* HMGB1*, and the noncoding RNA* LINC00426* [3].

Here, we describe a Caucasian patient with a* de novo* complex chromosomal rearrangement [t(7;13) and t(11;15)] including a 3–3.4 Mb microdeletion on 13q12.2q13.1, overlapping with the 13q12.3 microdeletion syndrome region. This subject displays the characteristic dysmorphic features highlighted in the recently reported cases, as well as psychomotor developmental delay and markedly delayed speech.

## 2. Clinical Report

**Figure 1:**
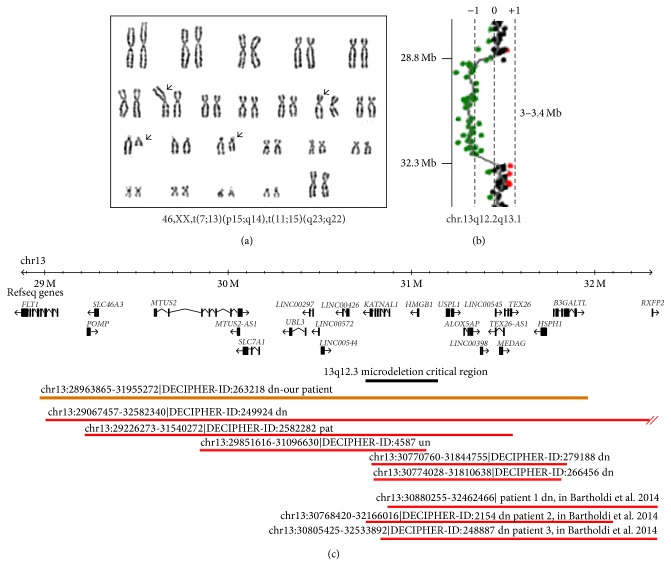
Karyotype, array-CGH analysis and schematic representation of the deleted region with reported DECIPHER cases. (a) The G-band karyotype of the patient. Black arrows indicate translocated chromosomes. (b) Chromosome 13 array-CGH results [arr 13q12.2q13.1 (28,875,081x2, 28,963,865-31,955,272x1, 32,313,799x2)dn]. (c) A scheme of the deleted region with distances in Mb and the Refseq genes are reported (GRCh37/hg19). The region deleted in our patient (code: 263218, orange bar) and the overlapping reported deletions (red bars) as shown by the Decipher database (http://decipher.sanger.ac.uk/, version 5.1) are reported. Above each bar the extension of the rearrangement and the mode of inheritance is reported (dn:* de novo*; pat: paternal origin, and un: unknown).

**Figure 2:**
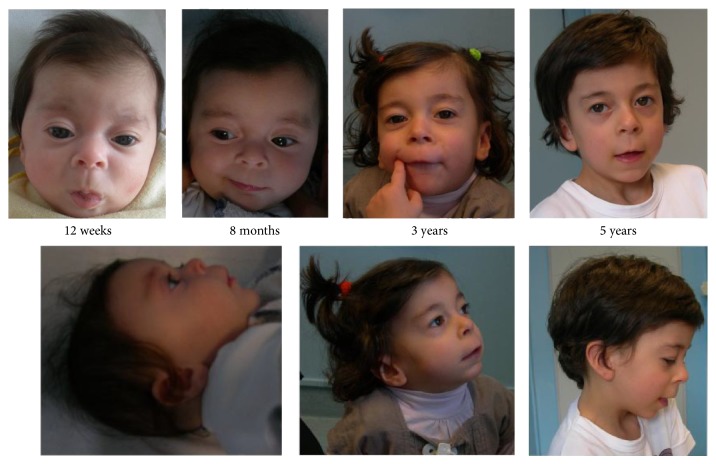
Proposita at 12 weeks, 8 months, 3 years, and 5 years old.

The proband was the third daughter of a 41-year-old mother and a 42-year-old father. Both parents and siblings were healthy. Prenatal ultrasounds did not reveal any foetal malformations. Prenatal karyotype analysis by standard GTG banding, performed due to advanced maternal age, showed a* de novo* double translocation [46,XX,t(7;13)(p15;q14),t(11;15)(q23;q22)] (Figure 1(a)); UPD was ruled out for chromosomes 7, 11, and 15. Birth occurred through elective caesarean section at the 38th week of gestation (APGAR 7/8). Birth parameters were at the 50th centile according to the Italian growth curves (length: 50 cm (50th cent); weight: 2.79 kg (50th cent); head circumference: 34.5 cm (50th cent)). We excluded cerebral malformations by brain ultrasound analysis and ocular defects by carrying out a* fundus oculi* exam. ECG examination showed a long QT (QTc: 470 ms), which was not reconfirmed at 17 days. Dysmorphisms included large wide set eyes, long philtrum, thin upper lip, and large ears (Figure 2). An angioma was present on the thorax and another was found on the top of the head.

From infancy to the last follow-up at 5 years old, growth parameters were consistently below target levels. At 5 years, the measured parameters of the child were as follows: height: 100 cm (3rd cent); weight: 13 kg (<3rd cent/−2.8 SD); head circumference: 49 cm (10th cent). She displayed psychomotor delay: at 8 months she was unable to sit unsupported and at 18 months, after physiotherapy treatment, she still required support for walking. Lallation began at 16 months and language development was markedly impaired (at 5 years she pronounced very few words that included phonological alterations). The Griffiths test performed at 20 months old revealed a mental age of 14.4 months.

Examination at 2 years old revealed a normal EEG but brain MRI showed mild hypomyelination of the subcortical regions and thinning of the* corpus callosum*. Urinary and plasmatic aminoacid screenings were normal.

Postnatal array-CGH 44 K (Agilent, Santa Clara, CA) performed at 1 year old identified a 3–3.4 Mb microdeletion on chromosome 13q12.2q13.1, close to the translocation breakpoint on chromosome 13 (Figure 1(b)). The deletion was shown to span from position 28,963,865 to 31,955,272 (minimal region) (NCBI Build 37/hg19), to contain 20 transcripts (15 coding genes), and did not overlap with common copy number variants (Database of Genomic Variants, http://projects.tcag.ca/variation/) (Figure 1(c)). The deletion was confirmed by real-time quantitative PCR assay designed to target exon 4 of the microtubule-associated tumour suppressor candidate 2 gene (*MTUS2*, NM_001033602.2). The same assay was used to confirm the* de novo* origin of this rearrangement.

## 3. Discussion

The 13q12.3 microdeletion syndrome was recently described in three patients presenting with intellectual disability, microcephaly, and eczema/atopic dermatitis [3]. Here, we describe a fourth patient with strikingly similar dysmorphic features, confirming the presence of a recognizable phenotype.

Common clinical features included reduced head circumference, triangular face, high frontal hairline, large ears, wide set eyes, fullness of eyelids, malar flattening, a prominent nose with underdeveloped* alae nasi* and low insertion columella, thin upper lip vermilion, and a pointed chin. Our patient had one episode of cutaneous rash, but a specific diagnosis of atopic dermatitis was not made. All patients have shown delayed speech development and moderate intellectual deficit. In three out of four patients recurrent upper airway respiratory infections were reported. Other features shared by the described patients (namely, recurrent vomiting, failure to thrive, allergies, abnormal vision, oligodontia, or truncal obesity) were not observed in our proband.

Several other deletions and duplications that partially or totally overlap the present one, which are associated with a particular phenotype, are reported in the Decipher database (https://decipher.sanger.ac.uk/) (Figure 1(c) and Table 1). In particular, four deletions are partially or totally included in the deletion of this case study (DECIPHER cases numbers 249924, 282282, 4587, 266456, and 279188) and these patients show intellectual disability, language delay, microcephaly, and facial dysmorphisms. Moreover, behavioural abnormalities similar to the ones described by Bartholdi et al. were reported in two cases (Table 1). The minimal shared region between these deletions and the deletion reported in our patient includes the three genes* KATNAL1* (MIM 614764),* LOC100188949*, and* HMGB1* (MIM 163905) [3]. A causal role can be easily suggested for* HMGB1* only, which encodes a ubiquitous nonhistone chromosomal protein expressed in brain (Allen Mouse Brain Atlas, http://mouse.brain-map.org/). This is a possible dosage-sensitive gene involved in the inflammatory response that may contribute to neuronal excitability and seizures [4, 5].

Three other genes within the deleted region in our patient, but not found to be involved in the patients investigated by Bartholdi et al., are associated with OMIM phenotypes: (i) UDP-Gal:beta-GlcNAc beta-1,3-galactosyltransferase-like (*B3GALTL*, MIM 610308), which is mutated in the autosomal recessive Peters plus syndrome (MIM 261540), characterized by anterior eye-chamber abnormalities, disproportionate short stature, and developmental delay [6]; (ii) arachidonate 5-lipoxygenase-activating protein (*ALOX5AP*, MIM 603700) gene, whose sequence variants confer increased susceptibility to stroke (MIM 603700) [7]; (iii) proteasome maturation protein (*POMP*, MIM 613386) gene, which is associated with the recessive phenotype keratosis linearis with ichthyosis congenita and sclerosing keratoderma (MIM 601952) [8]. The clinical features of these diseases are not clearly related to our patient's phenotype, in line with the recessive nature of the associated syndromes. Comparison of our patient with the four reported in Decipher did not suggest any phenotypic effect of the additional deleted genes in the 13q12.2q13.1 region. Moreover, we cannot exclude the occurrence of position effect or gene disruption at any of the breakpoints of this complex karyotype and the deletion span may contribute to the phenotypic differences. Thus, the gene(s) responsible for the phenotypic differences with reference to the individuals in the study of Bartholdi et al. presently remains elusive.

The relevance of the 13q12q13 deletion, currently supported by the phenotypic similarity and* de novo* deletion origin of the three DECIPHER cases, the three individuals reported by Bartholdi et al., and our affected subject will be confirmed by identifying additional cases, in particular those carrying point mutations in* HMGB1* or another gene in this region.

## Figures and Tables

**Table 1:** Phenotypic features of Decipher patients with a deletion that overlaps with our case.

Decipher code	263218 (our case)	249924	282282	4587	266456	279188	Patient 1 [3]	2154 Patient 2 [3]	248887 Patient 3 [3]
From (bp) To (bp) Size (Mb) Inheritance	28,963,865 31,955,272 (3.4) *De novo *	29,067,457 32,582,340 (3.51) *De novo *	29,226,273 31,540,272 (2.31) Paternal^*^	29,851,616 31,096,830 (1.25) Unknown	30,774,028 31,810,638 (1.04) *De novo *	30,770,760 31,844,755 (1.07) *De novo *	30,880,255 32,462,46 (1.58) *De novo *	30,768,420 32,166,016 (1.4) *De novo *	30,805,425 32,533,8 (1.73) *De novo *
Age (yrs)	5	15	11	?	2	0	19	12	12,5
Height	3rd cent					Growth ret.	3rd–10th cent	25th–50th cent	<3rd cent
Weight			Obesity				25th–50th cent	25th–50th cent	<3rd cent
Microcephaly	OFC: 10th cent		+	+		+	n.a.	OFC: 25th cent	−
Ears	Large			Large					Hearing loss
Wide set, large eyes	+			−			+	+	+
Puffy eyelids	+						+	+	+
Eyes, others	Normal vision					D.p.f	Hypermetropia	Hypermetropia	Hypermetropia
Narrow nasal bridge	+			+			+	+	+
Underdeveloped alae nasi	+						+	+	+
Low insertion columella	+			+			+	+	+
Thin vermilion upper lip	+						+	+	+
Oligodontia	−						+	−	+
Thorax	Mild pectus excavatum			Pectus excavatum					
Atopic dermatitis	One episode of cutaneous rash						+	+	+
Skin	Two haemangiomas			Spotty hyperpigm					
Others			Metatarsus adductus; hypogonadism	2-3 toe syndactyly	Cutaneous finger syndactyly		Hip dysplasia; cryptorchidism	Congenital hernia of diaphragm	Asymmetry legs
Intellectual deficit	+		+	+	+	+	+	+	+
Language delay	+		+		+	+	+	+	+
Behavioural abnormalities	−		ADHD	Hyperactivity			Hyperactivity	Hyperactivity	Hyperactivity
Hypotonia	+								
Neurological features			Hyperreflexia						

Note: ADHD: attention deficit hyperactivity disorder. n.a.: not available. del: deletion. Asterisk indicates that the father of patient 282282 was affected. hyperpigm: hyperpigmentation. D.p.f.: Downslent palpebral fissures; Phanotype of 249924 was not available; Growth ret.: growth retardation.

## Abbreviations

CGH: Comparative genome hybridization
CCR: Complex chromosomal rearrangements
UPL: Universal probe library
UPD: Uniparental disomy
ECG: Electrocardiogram
EEG: Electroencephalogram
MRI: Magnetic resonance imaging.
